# Enhanced putamen functional connectivity underlies altered risky decision-making in age-related cognitive decline

**DOI:** 10.1038/s41598-023-33634-w

**Published:** 2023-04-24

**Authors:** Ping Ren, Gangqiang Hou, Manxiu Ma, Yuchuan Zhuang, Jiayin Huang, Meiling Tan, Donghui Wu, Guozhi Luo, Zhiguo Zhang, Han Rong

**Affiliations:** 1 Department of Geriatric Psychiatry, Shenzhen Mental Health Center/Shenzhen Kangning Hospital, Shenzhen, Guangdong China; 2 Department of Radiology, Shenzhen Mental Health Center/Shenzhen Kangning Hospital, Shenzhen, Guangdong China; 3 Queensland Brain Institute, University of Queensland, St. Lucia, QLD Australia; 4 Department of Electrical and Computer Engineering, University of Rochester, Rochester, NY USA; 5 Department of Psychiatry, Shenzhen Mental Health Center/Shenzhen Kangning Hospital, Shenzhen, Guangdong China; 6 School of Biomedical Engineering, Health Science Center, Shenzhen University, Shenzhen, Guangdong China

**Keywords:** Cognitive ageing, Cognitive neuroscience, Decision

## Abstract

Risky decision-making is critical to survival and development, which has been compromised in elderly populations. However, the neural substrates of altered financial risk-taking behavior in aging are still under-investigated. Here we examined the intrinsic putamen network in modulating risk-taking behaviors of Balloon Analogue Risk Task in healthy young and older adults using resting-state fMRI. Compared with the young group, the elderly group showed significantly different task performance. Based on the task performance, older adults were further subdivided into two subgroups, showing young-like and over-conservative risk behaviors, regardless of cognitive decline. Compared with young adults, the intrinsic pattern of putamen connectivity was significantly different in over-conservative older adults, but not in young-like older adults. Notably, age-effects on risk behaviors were mediated via the putamen functional connectivity. In addition, the putamen gray matter volume showed significantly different relationships with risk behaviors and functional connectivity in over-conservative older adults. Our findings suggest that reward-based risky behaviors might be a sensitive indicator of brain aging, highlighting the critical role of the putamen network in maintaining optimal risky decision-making in age-related cognitive decline.

## Introduction

Risky decision-making is a crucial ability of processing risk information in the external environment, which is critical to survival and development. Aging-induced brain deterioration has been found to be associated with abnormal financial risk-taking behaviors, and often leads to suboptimal decision making^1–3^. Compared with young adults, older adults have been found to have a higher preference for certainty in reward-related probabilistic task, leading to over- or under-estimation of risk options^4,5^. Growing evidence has shown that age-related differences in risk taking vary considerably depending on decision scenarios and cognitive abilities^6,7^. In recent years, many studies focus on the alterations of reward-related risk behaviors and relative brain dysfunction in age-related neuropsychiatric diseases such as Alzheimer’s disease and Parkinson’s disease^8,9^. Besides, older adults often have more financial resources but lower cognitive ability relative to young adults, thus impaired risky decision-making renders them uniquely susceptible to financial exploitation in daily lives^10^. Therefore, understanding the neural substrates of altered risky decision-making in elderly population is critical for successful aging.

Balloon analogue risk task (BART) measures reward-related decision making, which has been successfully applied in studying reward approaching and punishment avoiding behaviors in different contexts^11–14^. Compared with young adults, older adults exhibited a significantly lower degree of risk tolerance and impaired BART performance^15,16^. In contrast, some studies reported that young and older adults do not differ in their risk strategies in the BART^17,18^. The inconsistency implies that age-induced alterations of risk decision-making are probably more complicated, and some underlying factors need to be taken into account in the risk behaviors of elderly population. For instance, Seaman et al. reported that older adults living in a retirement community were more risk-averse than their independent counterparts in the BART, showing the influence of residential choice on risky decision-making^19^. In the Iowa gambling task, older adults were divided into task-advantageous and -disadvantageous subgroups, showing divergent neurobiological aging trajectories involved in reward-based decision making^20,21^. In addition, a growing body of evidence has shown a unique group of older adults (“super-ager”) who demonstrate comparable cognitive performance to younger or middle-aged individuals^22–24^. Based on these findings, we speculated that complex behavior such as financial risk behavior is of great inter-individual variability in older adults, and might be unimpaired in some elderly individuals.

Functional magnetic resonance imaging (fMRI) helps researchers observe real-time brain activity during resting state or performing task non-invasively by measuring blood oxygenation level-dependent (BOLD) signal. Converging evidence in fMRI studies has shown that multiple regions in the prefrontal cortex (PFC) and striatum are involved in reward-related decision making^25–27^. A recent study has examined the reliability of brain activation patterns in response to risk-taking during the BART, showing that bilateral striatum such as putamen is associated with win outcomes^28^. Another study reported the frontal, striatal, and medial temporal areas as critical components in distinguishing risk-seeking and risk-aversive older adults^29^, in line with our prior study showing a decline of intrinsic putamen activity at the early stage of age-related cognitive impairment^30^. Additionally, putamen volume loss has been found in aging and age-related neurodegenerative diseases^31,32^. Therefore, these findings suggest that age-related alterations in risky decision-making is likely due to striatum-centered dopaminergic dysfunction, and the cortico-putamen connectivity may be critical for understanding the suboptimal strategies in older adults. So far, it is still under-investigated about the neural substrates of altered risk bias in elderly people using the BART paradigm. Using a task-related fMRI design, Yu et al. reported that young and older adults showed no behavioral difference in the BART, whereas striatal and ventral medial PFC (vmPFC) activation was predictive of behavioral risk-taking for young but not older adults^33^. Task-related neuroimaging data reveals activation in brain regions during processing reward/risk information, therefore, it is of great importance to examine the intrinsic brain connectivity associated with age-related alterations of risk-taking behaviors for examining the generalized neural underpinnings of risky decision-making.

In the current study, we investigated the intrinsic putamen functional network in modulating risk-taking behaviors in older adults. To better characterize the individual differences of risk behaviors in aging, a two-step clustering model was applied to further examine the behavioral heterogeneity in the young and elderly groups, respectively. The Two-Step clustering algorithm has been demonstrated with several advantages, like determining the number of clusters based on a statistical measure of fit rather than on an arbitrary choice, and analyzing atypical values (i.e., outliers)^34–36^. It was expected that risky decision-making would change significantly in some older adults but not in others regardless of cognitive decline. Furthermore, we speculated that the putamen functional network is critical for maintaining optimal risky decision-making in aging.

## Results

### Demographic and behavioral data

**Figure 1:**
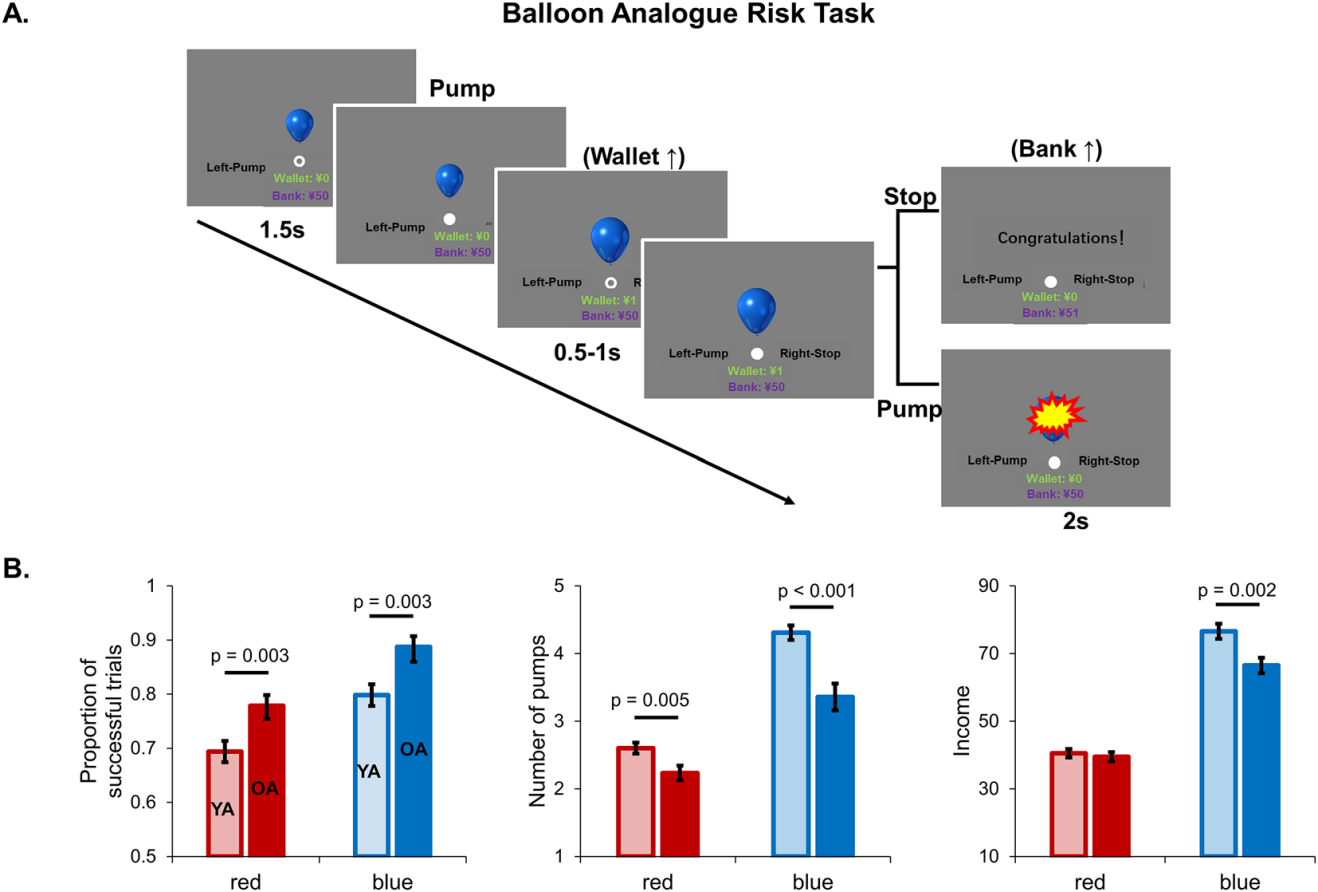
The BART paradigm and overall performance. (**A**) At the beginning of each trial, a fixation of white circle was displayed at the center of the screen for 1.5 s. After the fixation turned to a white disc, participants could increase monetary earnings in the “wallets” by inflating the balloon. The money in the “wallet” would be successfully transferred to the “bank” if participants stop pumping before balloon explosion, or it would be lost (feedback 2 s). The inter-trial interval was jittered pseudorandomly between 2 and 4 s. (**B**) Compared with young adults, older adults showed significantly higher ratio of successful trials, decreased average adjusted number of pumps, and lower income. Note: young adults, light color; older adults, dark color.

**Figure 2:**
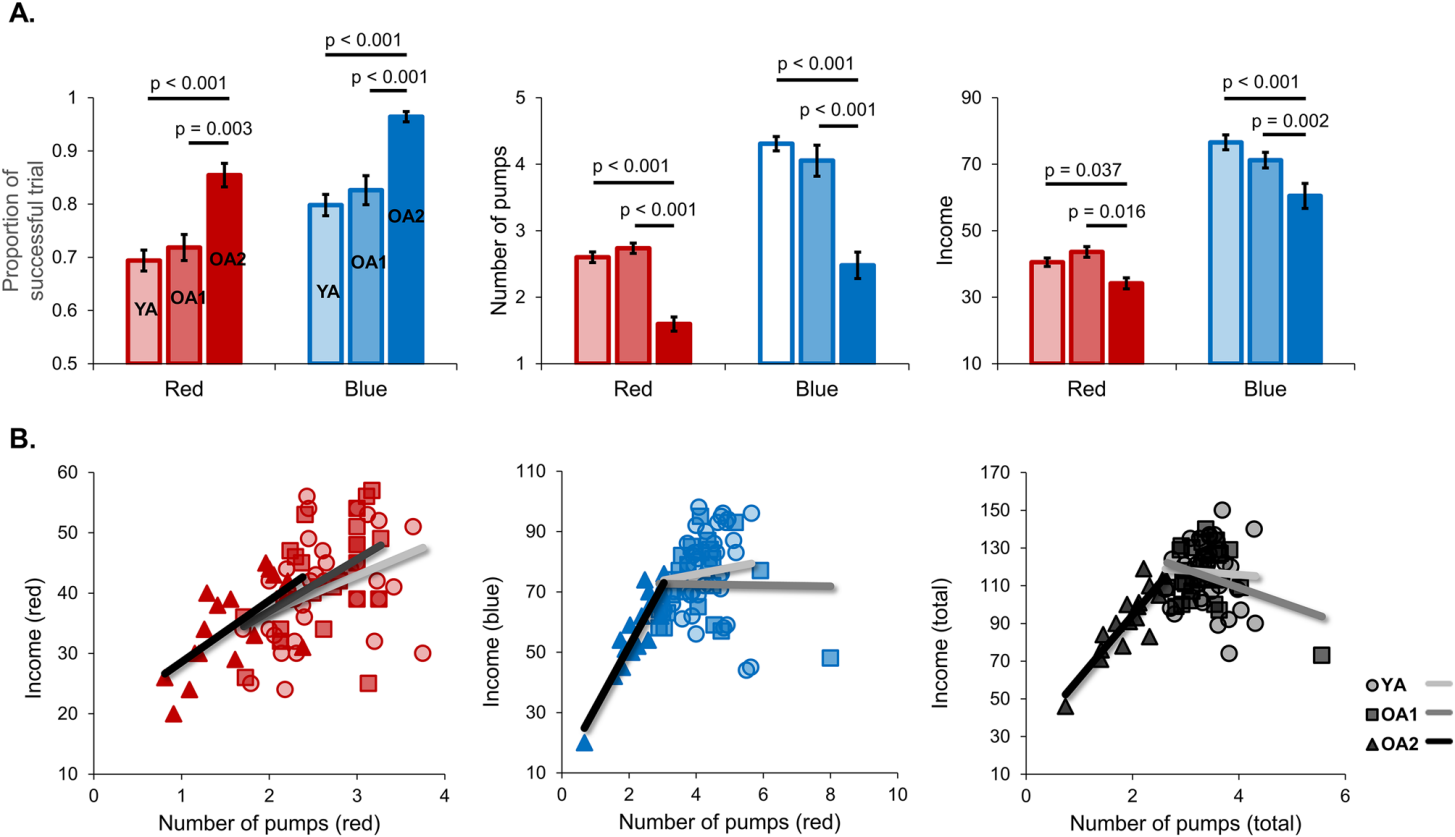
Behavioral performance of the BART. (**A**) Compared with the YA group, the OA1 group showed comparable task performance, including the ratio of successful trials, averaged adjusted number of pumps, and income for red and blue balloons. The OA2 group exhibited significantly poorer performance compared with other two groups (Bonferroni corrected *p* < 0.05). (**B**) A linear regression analysis was applied to examine the group differences in task performance. The relationship between income and adjusted number of pumps were significantly changed in the OA2 group for the blue and total conditions (middle and right plots), but not for the red condition (left plots). YA, light color; OA1,medium color; OA2, dark color.

Before the formal analysis, one older participant with total income (¥14) was excluded, due to extremely low income in the older group (< Mean-2.5SD). Eventually, 39 young adults and 40 older adults were involved in the formal analysis. Compared with young adults, older adults showed significantly worse task performance in the BART, except for the income in red balloon condition (Fig. 1B). Using the two-step cluster model, only older adults were successfully divided into two subgroups (OA1, n = 23; OA2, n = 17) with distinct task performances, supporting the large heterogeneity in older adults. One-way Analysis of variance (ANOVA) was applied to examine differences in demographic data and task performance across the three groups in Table 1. Notably, the OA1 group showed BART performance as good as the YA group, significantly better than the OA2 group (Table 1). In the generalized linear model, there were significant differences between the averaged adjusted number of pumps and income across the three groups in blue (Wald’s χ^2^ = 16.2, *p* < 0.001) and total conditions (Wald’s χ^2^ = 24.8, *p* < 0.001), but not in red condition (Wald’s χ^2^ = 1.0, *p* = 0.6) (Fig. 2).

**Table1:** Demographic, cognitive assessments and BART performance of participants.

	YA (n = 39)	OA1 (n = 23)	OA2 (n = 17)	*p* value
Age	25.0 ± 2.6	64.0 ± 4.6*	66.8 ± 5.6*	** < 0.001**
Male/female	20/19	9/14	8/9	0.65
Years of education	14.4 ± 2.6	12.0 ± 1.9*	11.8 ± 2.7*	**0.001**
MOCA	27.1 ± 1.6	22.4 ± 3.3*	21.8 ± 4.1*	** < 0.001**
MMSE	29.2 ± 1.1	27.8 ± 1.9*	27.5 ± 2.3*	**0.013**
BART performance				
Successful trials (%)	0.75 ± 0.09	0.77 ± 0.1	0.91 ± 0.06*,#	** < 0.001**
Adjusted pumps	3.5 ± 0.4	3.4 ± 0.6	1.9 ± 0.5*,#	** < 0.001**
Income	117.1 ± 16.8	114.4 ± 15.0	91.8 ± 18.0*,#	** < 0.001**

Data are presented as means ± standard deviations for the YA, OA1, and OA2 groups. One-way ANOVA was applied to examine the differences among the three groups. *Post-hoc* analysis was used to examine the differences between each pair of groups with Bonferroni corrected *p* < 0.05 (*, *p* < 0.05 compared with the YA; #, *p* < 0.05, compared with the OA1). Abbreviations: BART, balloon analogue risk task; MOCA, Montreal Cognitive Assessment; MMSE, Mini Mental State Examination; OA, older adults; YA, young adults.

Significant values are in [bold].

### Group differences in functional connectivity

**Figure 3:**
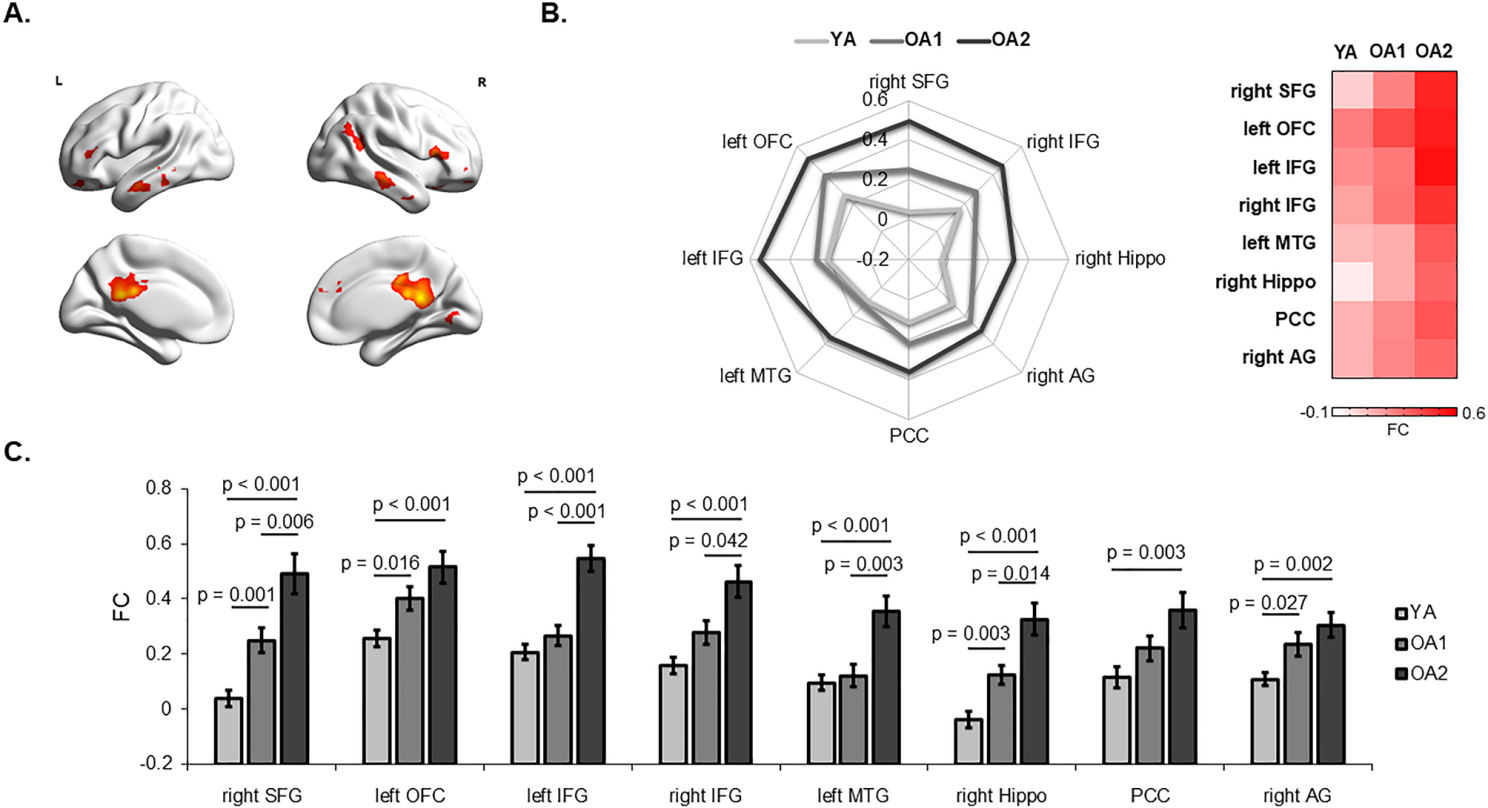
The group differences in the putamen functional network. (**A**) The ANCOVA was applied to examine the alterations of the putamen network in older adults, controlled for education, brain atrophy, and head motion. The functional connectivity of multiple brain regions was significantly changed in the two old groups, including the PFC, temporal and parietal lobe. (**B**) The radar map showed the pattern of putamen connectivity in the YA, OA1, and OA2 groups. Relative to young adults, the connectivity pattern was similar in the OA1 group, but largely changed in the OA2 group. (**C**) The peak values of connectivity strength within the significant regions were extracted and plotted for the three groups (Bonferroni corrected *p* < 0.05). Abbreviations: AG, angular gyrus; Hippo, hippocampus; MNI, Montreal Neurological Institute; IFG, inferior frontal gyrus; MTG, middle temporal gyrus; OFC, orbital frontal cortex; PCC, post cingulate cortex; SFG, superior frontal gyrus.

In the Analysis of covariance (ANCOVA), the putamen network showed significant age-related changes in multiple brain regions, including the right superior frontal gyrus (SFG), left orbitofrontal cortex (OFC), bilateral inferior frontal gyrus (IFG), left middle temporal gyrus (MTG), right hippocampus, posterior cingulate cortex (PCC) and right angular gyrus (AG) (Table 2 and Fig. 3A). In addition, the left and right putamen networks was calculated and compared between the three groups respectively, and showed consistent patterns of connectivity (Supplementary Table 1 and Supplementary Fig. 2). In Fig. 3, a radar map shows altered patterns of the putamen connectivity between the YA, OA1, and OA2 groups. In Fig. 3B, the connectivity strength of eight brain regions was organized into a connectivity vector for each group. Pearson correlation was applied to calculate SI for each paired group, showing significantly high similarity between the YA and OA1 groups (SI = 0.82, *p* = 0.042), but not between the YA and OA2 groups (SI = 0.63, *p* = 0.096) or between the OA1 and OA2 groups (SI = 0.70, *p* = 0.077), with the false discovery rate (FDR) correction (*p* < 0.05). In addition, Spearman correlation was performed to confirm the similarity between groups, showing significantly high similarity between the YA and OA1 groups (SI = 0.83, *p* = 0.033), between the OA1 and OA2 groups (SI = 0.81, *p* = 0.033), but not between the YA and OA2 groups (SI = 0.64, *p* = 0.096) (FDR corrected *p* < 0.05).

**Table 2:** The putamen network was changed and correlated to risk behaviors across groups.

Region	Peak (F value)	Cluster (voxels)	MNI coordinates	Adjusted pumps (*r*)
Right SFG	11.5	171	15, 54, 21	− 0.39*
Left OFC	15.5	423	**− **24, 45, -21	**− **0.27
Left IFG	14.6	265	**− **45, 24, 12	− 0.42*
Right IFG	16.8	622	54, 21, 12	− 0.36*
Left MTG	13.6	453	**− **60, 0, **− **21	− 0.47*
Right hippocampus	20.0	459	39, **− **27, **− **9	− 0.39*
PCC	24.2	1522	3, **− **42, 18	**− **0.25
Right AG	13.8	271	45, **− **60, 36	**− **0.25

Significant group differences in the putamen network were examined using ANCOVA, controlled for education, head motion, and brain atrophy (*p* < 0.05, GRF correction). Partial correlation was applied to examine the relationships between connectivity strength and adjusted pumps in the BART, controlled for education, head motion, and brain atrophy (FDR corrected *p* < 0.05 for multiple comparison). Abbreviations: AG, angular gyrus; MNI, Montreal Neurological Institute; IFG, inferior frontal gyrus; MTG, middle temporal gyrus; OFC, orbital frontal cortex; PCC, post cingulate cortex; SFG, superior frontal gyrus.

Significant values are in [bold].

### Mediation analysis

**Figure 4:**
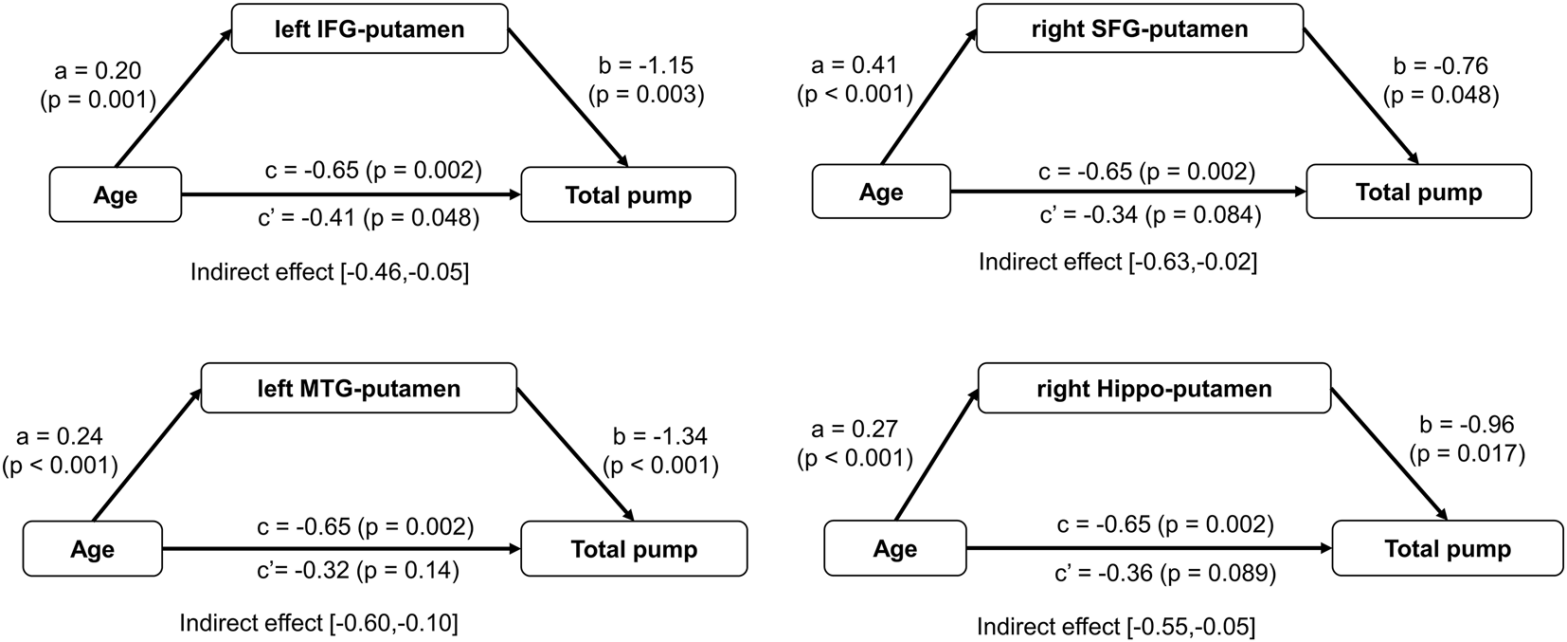
Age-effect on risk behaviors mediated by the putamen network. Mediation analyses showed that age influenced adjusted number of pumps mainly through the fronto-putamen and temporal-putamen connectivity.

A Mediation model was applied to examine the role of putamen functional network in modulating the relationship between age and risk-taking behavior, controlled for education, brain atrophy, and head motion. Of the eight significant brain regions, the right SFG, left IFG, right IFG, left MTG and right hippocampus were selected as a possible mediator because they were correlated to both age and adjusted number of pumps (Table 2). In Fig. 4, the relationships between age and averaged number of total pumps were significantly mediated by the connectivity of the putamen connectivity. Notably, the relationship between age and adjusted pumps was fully mediated by the left IFG-putamen connectivity.

### Group differences in gray matter volume

**Figure 5:**
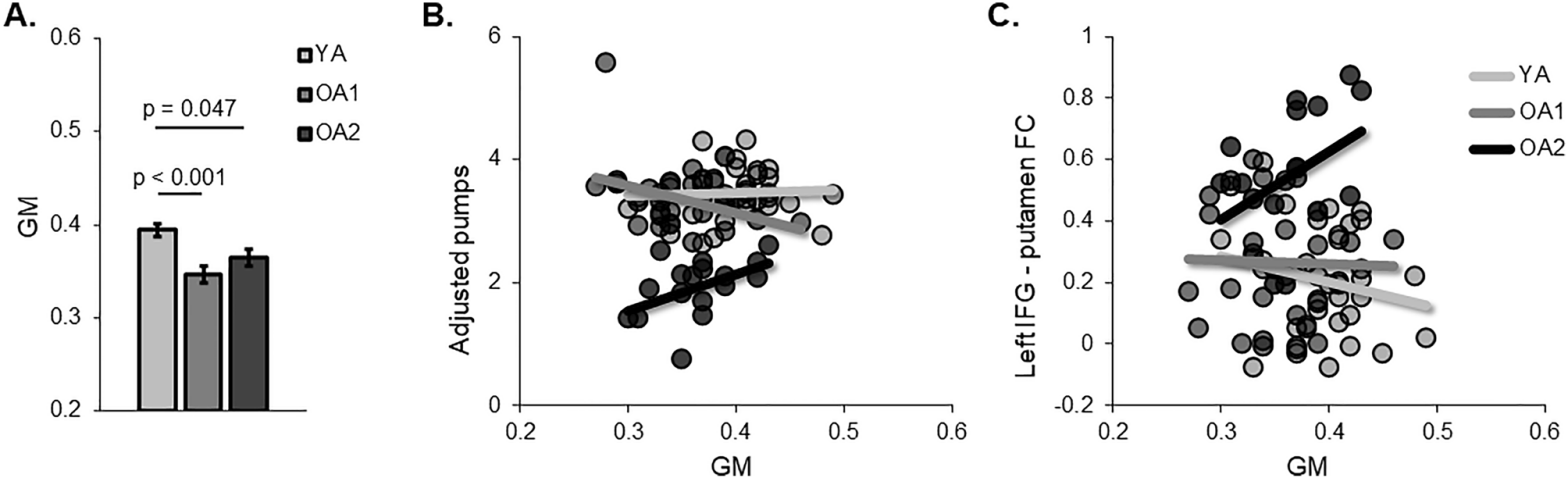
Group differences in the putamen GM and its relationship with risk behaviors. (**A**) The putamen gray matter volume was significantly decreased in the two old groups, compared with the young group (Bonferroni corrected *p* < 0.05). (**B**) A linear regression analysis was applied to examine the group differences in the relationships between GM and average adjusted number of pumps, showing significantly changed correlation in the OA2 group. (**C**) The relationship between GM and left IFG-putamen connectivity was significantly changed in the OA2 group.

Compared with young adults, the OA1 and OA2 groups showed significantly different GM of the putamen (Fig. 5A). In the generalized linear model, there was a significant difference in the relationships between the averaged adjusted number of pumps and putamen GM across the three groups (Wald’s χ^2^ = 10.8, *p* = 0.005), showing changed relationship in the OA2 group (Fig. 5B). Likewise, a significant difference was observed in the relationships between the left IFG-putamen connectivity and putamen GM across the three groups (Wald’s χ^2^ = 6.4, *p* = 0.042), showing changed relationship in the OA2 group (Fig. 5C). In addition, non-interested covariates were controlled in the model, including years of education, brain atrophy, and head motion. There was no significant group effect on the relationships between putamen GM and other connections.

## Discussion

The current study, for the first time, investigated the relationship between changed risky decision-making and the putamen functional network in older adults by combining the BART behaviors and resting-state fMRI measurements. Based on the task performance, two subgroups of older adults were identified, showing young-like and over-conservative decision strategies respectively. The functional connectivity of the putamen network was found significantly different between the three groups, showing remarkably increased connectivity strength in over-conservative older adults (OA2 group). Notably, age-effects on risk-taking behaviors were mediated via the putamen functional network. Furthermore, the anatomical changes of the putamen were observed to be associated with altered decision strategy and functional connectivity in over-conservative elderly group.

Aging-induced brain deterioration affects cognitive function in multiple domains, particularly complex behaviors such as risk-taking behaviors^3^. Compared with young adults, older adults exhibited a greater preference for sure gains and greater avoidance of sure losses, suggesting that they may weigh certainty more heavily than younger adults^4^. Consistently, we found overall decreased adjusted number of pumps and higher proportion of successful trials in older adults, supporting the theory of risk avoidance in aging. Furthermore, older adults with distinct task strategies were distinguished by using the two-step classification model. Rather than an arbitrary choice, here the two-step clustering model determined the elderly subgroups based on a statistical measure of fit, showing that a subset of older adults still performed optimal decisions. In the generalized linear model, the relationship between the adjusted number of pumps was significantly less in the OA2 group, suggesting an over-conservative risk-taking strategy. Although individuals in the OA1 group showed significantly declined cognitive ability, their BART performances were as good as young adults. Our results imply that risk-taking behaviors might be a sensitive indicator for assessing brain aging. Notably, processing speed has been found to be compromised in aging, and mediates age-related differences in BART performance^12,16^. Since MoCA/MMSE scores measure individual’s overall cognitive ability, the similarity and difference of BART performance between the YA, OA1 and OA2 groups might be associated with a specific cognitive domain such as processing speed. In addition, the current analyses included several older adults with low MoCA/MMSE scores, who might have potential cognitive deficits or high risk of mild cognitive impairment (MCI). However, it is still debatable whether normal aging and MCI are significantly different in reward-based decision making^37–39^, which needs to be investigated in the future. Taken together, the alteration of risk-taking behaviors in aging is more complicated than expected, showing that some older adults still maintain optimal financial decision making irrespective of cognitive decline. These findings would be helpful for understanding the negative behavioral results in previous studies, supporting the previous findings on supernormal older adults or super-agers^24,40^.

Compared with younger adults, multiple brain regions involved in the putamen network exhibited enhanced connectivity strength in older adults. Consistently, Fjell et al. found a positive age effect on cortico-subcortical functional networks, showing increased putamen-based connectivity in normal aging^41^. Specifically, we observed that connectivity strength between the PFC subregions (i.e., the right SFG, left and right IFG) and the putamen was significantly increased and negatively correlated with number of pumps in aging, in line with previous findings of the fronto-striatal network in modulating reward/risk information^42^. Using non-invasive brain stimulation, previous studies have reported that transcranial electrical stimulation over dorsolateral prefrontal cortex reduced risk-taking behaviors^43,44^. Therefore the hyperactivation of intrinsic fronto-putamen connectivity might indicate a strengthened inhibitory effect of the PFC in risk-seeking motivation, which induces an age-related constriction in sensitivity to monetary reward. The connectivity between putamen and middle temporal/hippocampal areas may indicate disrupted reward-related memory or reinforcement learning during risky decision-making^45^. As core hubs in the default mode network (DMN), the PCC and AG in the putamen network were found significantly changed as well, suggesting an altered striatal-DMN connectivity in aging. Notably, the connectivity strength was considerably increased in the over-conservative elderly group, while moderately increased in the young-like elderly group. Compared with young adults, the young-like group showed higher similarity of connectivity pattern than the over-conservative group. Consistent with our findings in the young-like elderly group, a recent study reported no age-related difference in adjusted pumps in the BART between young and older adults, suggesting a possible compensatory effect of increased neural coupling between the ventral medial PFC and anterior insula^17^. Additionally, some studies argued that the general non-efficient overactivation is a reflection of reduced dopamine functions in older age^46^. Based on these findings, we speculate that whether enhanced connectivity in older adults is a reflection of compensatory effect or reduced efficiency may depend on elderly groups with different cognitive abilities.

In the Mediation analysis, the putamen network was found as a mediator in the relationship between age and risk-taking behaviors. Specifically, age influences the risky decision-making through the connectivity of the left IFG, right SFG, left MTG and right hippocampus within the putamen network, suggesting critical roles of the fronto-striatal and temporal-striatal network in the altered risk-taking strategy of older adults. In line with our findings, converging evidence has demonstrated that fronto-striatal circuit is closely related to reinforcement learning in reward-related tasks^45^. Meanwhile, abnormal activation of the fronto-striatal network has been found associated with impaired cognitive modulation process in healthy older adults^47,48^, suggesting an altered cognitive control in dealing with financial risks. Another possibility is that age effect on risk-taking behavior (i.e., decreased number of pumps) might be due to reduced intrinsic motivation or cognitive fatigue^16,49^, which have been found closely associated with the putamen network^1,47^. In addition, the hippocampus has been reported to contribute to temporal associative processing in memory, whereas the striatum contributes to temporal motor preparation and reward anticipation^50,51^. As a typical characteristic of aging, the dysfunctional hippocampal/temporal-putamen connectivity implies a possible impairment of reward-based learning in older adults^46^. In the anatomical analysis of the putamen, the gray matter volume decreased significantly in elderly group, and showed positive correlation with number of pumps and left IFG-putamen connectivity in over-conservative older adults. The abnormal relationships might indicate an enhanced frontal regulation effect on the striatum, inducing a conservative strategy in dealing with reward/loss information. Notably, although the current analyses revealed the putamen network underpinning altered risk-taking behaviors in aging, it might not be the unique neural circuit. Other brain regions, such as insula and vmPFC^17,33^, have also been found to be involved in reward-based decision making, which should be examined in future research. Since the current analyses were based on healthy young and older populations, caution should be made when interpreting and extending the conclusions to other populations (e.g., Parkinson disease).

There are several limitations that need to be admitted. First, two elderly subgroups were artificially defined based on task performances using two-step clustering model. Although the two elderly subgroups represented considerably different risky behaviors and corresponding connectivity patterns, the clustering algorithm might not be optimal due to small sample size and task dependence. It is necessary to validate our findings in a large cohort with different clustering models in future research. Second, previous research has reported that individuals with a higher level of education may take more risks in financial activities^52^. Although years of education has been controlled as a covariate in our analysis, it should be validated in future studies with balanced educational level. Third, although the current study confirmed the considerable differences of risk-taking behaviors between young and older adults in line with previous findings, the cross-sectional design may result in a potential spurious correlations in mediational analyses (particularly designs with extreme age groups)^16,53^. Further analysis were performed to examine the correlations between BART performance and putamen connectivity for the young and older groups, separately (Supplementary Table 2). The patterns of correlations were in line with our hypothesis that older individuals’ suboptimal behaviors were associated with altered putamen connectivity. However, future research should examine the trajectory of decision making changing with age in a longitudinal design. At last, older adults with young-like decision strategy might be due to a compensatory effect of enhanced putamen connectivity, which is difficult to interpret using resting-state imaging data in the current analyses. Therefore, task-related fMRI study would be helpful for examining the relationship between altered risk-taking behaviors and neural efficiency.

## Conclusions

In summary, the current study found that altered risky decision-making was closely associated with enhanced putamen functional connectivity in aging. In particular, the fronto-putamen and temporal-putamen networks mediated the relationship between age and decision strategies. Our findings suggest that reward-based risky behaviors might be a sensitive indicator of brain aging, highlighting the critical role of the putamen network in maintaining optimal risky decision-making in age-related cognitive decline.

## Material and methods

### Participants

Eighty-six healthy participants were recruited from multiple communities in Shenzhen, including 41 young adults (YA) aged 20–31, and 45 older adults (OA) aged 58–80. We recruited participants by sending out flyers and advertisements in communities. In group fMRI analysis, Geuter et al. recently reported that sample sizes of 40 are adequate to identify regions with large effect sizes (Cohen’s d > 0.8), and sample size of 80 can identify regions with medium effect sizes (0.5 < d < 0.8)^54^. Therefore, a total sample sizes of 86 (more than 40 participants in each group) might be sufficient to detect large effect sizes. All participants were right-handed determined by the Edinburgh handedness inventory, have adequate visual and auditory acuity for testing by self-report. Exclusion criteria included (1) a history of diagnosed neurological or psychiatric diseases (i.e., major depression, anxiety, cerebrovascular disease); (2) a clinical diagnosis of MCI or dementia; and (3) MRI contraindications (i.e., metallic implant, claustrophobia, pacemaker). This study was performed in accordance with the Declaration of Helsinki and had approved by the Ethics Committee of Shenzhen Kangning Hospital (No. 2020-K2004-01). Each participant was required to sign a written informed consent form after a full written and verbal explanation of the study. Cognitive function was assessed by using the Montreal Cognitive Assessment (MoCA) and Mini Mental State Examination (MMSE). For MMSE, the scores were ranged 25–30 in the young group, and 20–30 in the older group. For MoCA, the scores were ranged 24–30 in the young group, and 16–29 in the older group.

### Balloon analogue risk task and performance analysis

In the BART, participants were presented with a virtual balloon and required to press the button in their left hand to pump the balloon (Fig. 1A). With the balloon inflating, monetary earnings in the “wallet” were increased, as well as the risk of explosion. Participants could continue inflating the balloon to increase money in the “wallet”, or stop by pressing the button in their right hand. The money in the ”wallet” would be successfully transferred to the ”bank” if participants stopped inflating before the balloon explosion, otherwise the money in the “wallet” would be lost. The wager was ¥1 per pump, and the probability of explosion monotonically increased with the number of inflations (Supplementary Table 3). Two colored balloons with different probability of explosion were used in the task: red balloon with a high risk of explosion (maximal number of pumps was 7) and blue balloon with a low risk of explosion (maximal number of pumps was 15). Participants made response as soon as the central white circle changed to a white disc (the time interval was jittered). After each trial, a feedback screen with “Congratulations!” or “Explosion” would be displayed for 2 s, then the next trial would begin after a 2–4 s inter-trial interval. Participants were required to collect money as much as possible by learning the covert rules over time. After completing the entire task, participants would be paid cash (¥50-¥200) based on their total income. The entire task lasts 10 min, with approximately 80 trials determined by individuals’ responses. The participants were required to complete the BART during a task-related fMRI session in the scanner after the resting-state scanning. Since the task-related data analysis was beyond the purpose of the current study and was used in another project, we didn’t include those data in the current study.

In each colored balloon condition, the proportion of successful trials (the number of unexploded trials divided by the total number of trials), adjusted number of pumps (averaged number of pumps in successful trials), and income (i.e., final monetary earnings in the bank) were used to measure risk-taking strategy for each participant. To better describe the alterations of risky decision-making in aging, two-step cluster model was applied to further subdivide young and older adults. The Two-Step clustering algorithm has been found well suited for multimodal data such as clinical and MRI data, representing cognitive heterogeneity across groups^35,55^. Briefly, the first step (pre-clustering) is to pre-cluster the individuals using a sequential-clustering method. In the second step (clustering), the pre-clusters are automatically grouped into optimal number of clusters based on a statistical measure of fit (e.g., Schwarz Bayesian Information Criterion). Using the proportion of successful trials, adjusted number of pumps and income (both red and blue balloons) as classifiers, the model successfully divided older adults into two subgroups: the OA1 group with advantageous decisions and OA2 group with disadvantageous decisions. The distributions of BART performance were displayed for the two groups in Supplementary Fig. 1. The young group could not be divided into subgroups, suggesting larger individual differences in aging. Then the following analyses were conducted based on three groups YA, OA1, and OA2. In addition, total proportion of successful trials, total adjusted number of pumps and total income were calculated regardless of red and blue conditions to do correlation and mediation analyses. For each age group, individuals with extremely low or high total income (2.5 standard deviation away from the mean) were defined as outliers and removed from the formal analyses.

### Imaging data acquisition

Imaging data were acquired on a 3.0 T MRI system (Discovery MR750 System, GE Healthcare) with an eight-channel phased-array head coil. The T1-weighted structural images were acquired by using a three-dimensional brain volume imaging sequence that covered the whole brain (repetition time (TR) = 6.7 ms, echo time (TE) = 2.9 ms, flip angle = 7 degrees, matrix = 256 × 256, slice thickness = 1 mm, 196 slices). Then the rs-fMRI data were acquired using gradient-echo echo-planar imaging sequence with the following parameters: TR = 2000 ms, TE = 25 ms, flip angle = 90 degrees, matrix = 64 × 64, and voxel size = 3.4 × 3.4 × 3.2 mm^3^, and 48 axial slices. For each participant, the resting-state fMRI (rs-fMRI) scan duration was 300 s with 150 volumes. During the resting-state scanning, the participants were required to open their eyes and relax without falling asleep. After the resting-state fMRI session, participants were required to perform the BART in a task-related fMRI session. However, only resting-state imaging data were involved in the current study.

### Imaging data processing

The resting-state imaging data were preprocessed using DPABI_v4.3 (https://rfmri.org/dpabi)^56^ based on SPM (http://www.fil.ion.ucl.ac.uk/spm/). For each participant, the first 10 volumes were excluded to obtain steady-state tissue magnetization. The remaining 140 volumes were corrected for slice timing and head motion, co-registered to their own structural images, and normalized to the Montreal Neurological Institute (MNI) standard space. Then the functional images were resampled to 3 × 3 × 3 mm, and smoothed with a Gaussian kernel (FWHM = 6 mm). After preprocessing, 2 young adults and 4 older adults were removed from the formal analysis due to head motion greater than 2 mm or 2 degrees. In addition, given the commonality of head motion in older adults and its confounding effect on resting-state functional connectivity^57^, the mean displacement of head motion was computed as follows: displacement = square root (x^2^ + y^2^ + z^2^) (x, left/right; y, anterior/posterior; z, superior/inferior), and controlled as a covariate in the following group analyses.

### Seed-based putamen functional connectivity

Before functional connectivity analysis, linear detrending was used to reduce the influence of MRI equipment, and a bandpass filter (0.01–0.1 Hz) was applied to reduce non-biological signals (e.g., respiratory and aliased cardiac signals). Then nuisance covariates were regressed out at the individual level, including 24 head motion parameters, white matter signal, and cerebrospinal fluid signal. To generate the striatal functional network, the putamen were defined by combining left and right putamen templates based on the Automated Anatomical Labeling (AAL) atlas^58^. The averaged BOLD time series within the putamen were extracted to correlate with that of each voxel in the entire brain. For each participant, the correlation coefficient map was Fisher's z transformed. ANCOVA was applied to examine the group difference of functional connectivity among the YA, OA1 and OA2 groups, controlled for years of education, brain atrophy, and head motion. The resulting statistical maps were corrected for multiple comparisons at *p* < 0.05 using Gaussian random fields (GRF) correction with voxel level *p* < 0.001 and cluster level *p* < 0.05. The peak value of functional connectivity was extracted within each significant brain region to do the following correlation and mediation analyses. Furthermore, in order to compare the connectivity patterns among the YA, OA1 and OA2 groups, the extracted connectivity values of all significant regions were organized into a connectivity vector for each group. Then the similarity level of connectivity patterns was defined as the similarity index (SI) by calculating the Pearson correlation coefficient between each pair of groups.

### Gray matter volume analysis

Voxel-based morphometry (VBM) was performed to generate a whole-brain gray matter map. The structural image of each participant was segmented into gray matter, white matter, and cerebrospinal fluid. Then, a gray matter template was generated through an iterative nonlinear registration using the ‘Diffeomorphic Anatomical Registration using Exponentiated Lie algebra’ (DARTEL), a toolbox with a fast diffeomorphic registration algorithm^59^. Based on the AAL atlas, individual’s gray matter (GM) volume of the bilateral putamen was extracted for the following group analysis. For each participant, averaged whole-brain gray matter was extracted for controlling age-induced brain atrophy.

### Other statistical analysis

Other statistical analyses were conducted in the Matlab 2014b and Statistical Product and Service Solutions (SPSS) V22. One-way ANOVA was applied to examine the group differences in age, years of education, MoCA, MMSE and BART performance, and a post hoc test was used for a pairwise comparison with Bonferroni correction (*p* < 0.05). Partial correlation analysis was applied to examine the relationship between task performance and functional connectivity, controlled for years of education, brain atrophy, and head motion. The mediation analysis was performed to examine the influence of the putamen network in the relationship between age and task performance (number of bootstrap samples = 5000) using the PROCESS macro in the SPSS^60^. In the mediation model, path a represents the relationship between independent variable X and mediator, and path b represents the relationship between mediator and dependent variable Y. The chief estimated path is the total effect of X on Y (i.e., path c). This path comprises the direct effect of X on Y after controlling for the (i.e., path c’) and the indirect effect of X on Y through the mediator (i.e., path a × b). After accounting for the mediator, the mediation effect was determined by assessing whether there was a significant difference between the total effect (i.e., path c) and direct effect (i.e., path c’). In the current study, X, Y, and mediator were age, adjusted pumps, and putamen connectivity, respectively. In addition, data of age and education were ln-transformed to reduce data skewness in the mediation analysis.

## Supplementary Information

Supplementary Information.

## Data Availability

The data generated by this study are undergoing additional analyses. The data used in this study is available from the corresponding author upon request.
