# Supplementary material for: Enhanced putamen functional connectivity underlies altered risky decision-making in age-related cognitive decline
Source: Sci Rep. 2023 Apr 24;13:6619. doi: 10.1038/s41598-023-33634-w (PMC10126002; doi:10.1038/s41598-023-33634-w)

**Supplementary Table 1. The group differences in the left and right putamen networks.**

| **Region** | **Peak (F value)** | **Cluster (voxels)** | **MNI coordinates** |
| --- | --- | --- | --- |
| *Left putamen network* |  |  |  |
| Right SFG | 11.8 | 142 | 9, 27, 39 |
| Left IFG | 15.9 | 742 | -45, 24, 12 |
| Left MTG | 12.3 | 404 | -60, 0, -21 |
| Right Hippocampus | 18.5 | 340 | 39, -27, -12 |
| Right PCC | 25.3 | 872 | 3, -42, 18 |
| Right AG | 11.8 | 182 | 45, -60, 36 |
| Right Cuneus | 15.0 | 318 | 6, -99, 0 |
| *Right putamen network* |  |  |  |
| Right Hippocampus | 18.4 | 1416 | 39, -27, -9 |
| Left MTG | 13.1 | 384 | -57, 6, -24 |
| Right PCC | 20.6 | 1426 | 9, -45, 24 |
| Right AG | 13.4 | 316 | 45, -60, 36 |

Significant group differences in the putamen network were examined using ANCOVA, controlled for education, head motion and brain atrophy (p < 0.05, GRF correction). Abbreviations: AG, angular gyrus; MNI, Montreal Neurological Institute; IFG, inferior frontal gyrus; MTG, middle temporal gyrus; PCC, post cingulate cortex; SFG, superior medial frontal gyrus.

**Supplementary Table 2. The putamen connectivity correlated to risk behaviors for the young and older groups.**

| **Region** | **Adjusted pumps (r)**  **YA** | **Adjusted pumps (*r*)**  **OA** |
| --- | --- | --- |
| right SFG | 0.02 | **-0.38 *** |
| left OFC | 0.02 | -0.20 |
| left IFG | 0.09 | **-0.50 *** |
| right IFG | 0.10 | **-0.41 *** |
| left MTG | -0.11 | **-0.49 *** |
| right hippocampus | -0.16 | **-0.45 *** |
| PCC | 0.04 | -0.23 |
| right AG | 0.15 | -0.24 |

Partial correlation was applied to examine the relationships between connectivity strength and adjusted pumps for the young and older groups, controlled for education, head motion, and brain atrophy (FDR corrected p < 0.05 for multiple comparison). Abbreviations: AG, angular gyrus; MNI, Montreal Neurological Institute; IFG, inferior frontal gyrus; MTG, middle temporal gyrus; OFC, orbital frontal cortex; PCC, post cingulate cortex; SFG, superior frontal gyrus; YA, young group; OA, older group.

**Supplementary Table 3. The risk of explosion for red and blue balloons.**

| **Number of inflation** | **Probability of explosion**  **(Red)** | **Probability of explosion**  **(Blue)** |
| --- | --- | --- |
| 1 | 0.00 | 0.00 |
| 2 | 0.06 | 0.01 |
| 3 | 0.12 | 0.02 |
| 4 | 0.24 | 0.06 |
| 5 | 0.42 | 0.08 |
| 6 | 0.58 | 0.12 |
| 7 | 0.74 | 0.18 |
| 8 | / | 0.24 |
| 9 | / | 0.32 |
| 10 | / | 0.42 |
| 11 | / | 0.52 |
| 12 | / | 0.60 |
| 13 | / | 0.70 |
| 14 | / | 0.80 |
| 15 | / | 0.90 |

For each color balloon, the probability of explosion monotonically increased with the number of inflations. The number of inflations ranged from 1 to 7 in red balloon, and from 1 to 15 in blue balloon.

**Supplementary Figure 1. The distributions of task performance in the OA1 and OA2 groups.** In the two-step clustering model, older adults were divided into two subgroups based on six classifiers (proportion of successful trials, adjusted number of pumps and income in red and blue balloons). The distribution plots show the overall BART performance of the OA1 and OA2 groups, respectively.


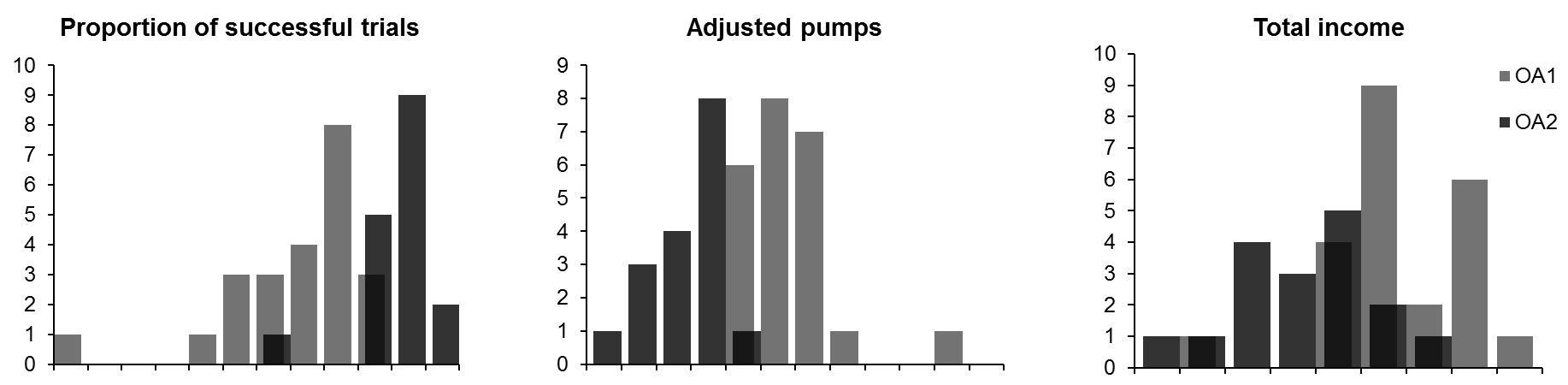


**Supplementary Figure 2. The group differences in the left and right putamen functional network. A)** For the left and right putamen networks, the ANCOVA was applied to examine the differences of the putamen network in older adults, controlled for education, brain atrophy, and head motion. Multiple brain regions within the putamen networks were observed significantly different in the two old groups, including the PFC, temporal and parietal lobe. Consistent with the findings in bilateral putamen network, the connectivity strength was significantly higher in over-conservative older adults relative to young-like older adults.


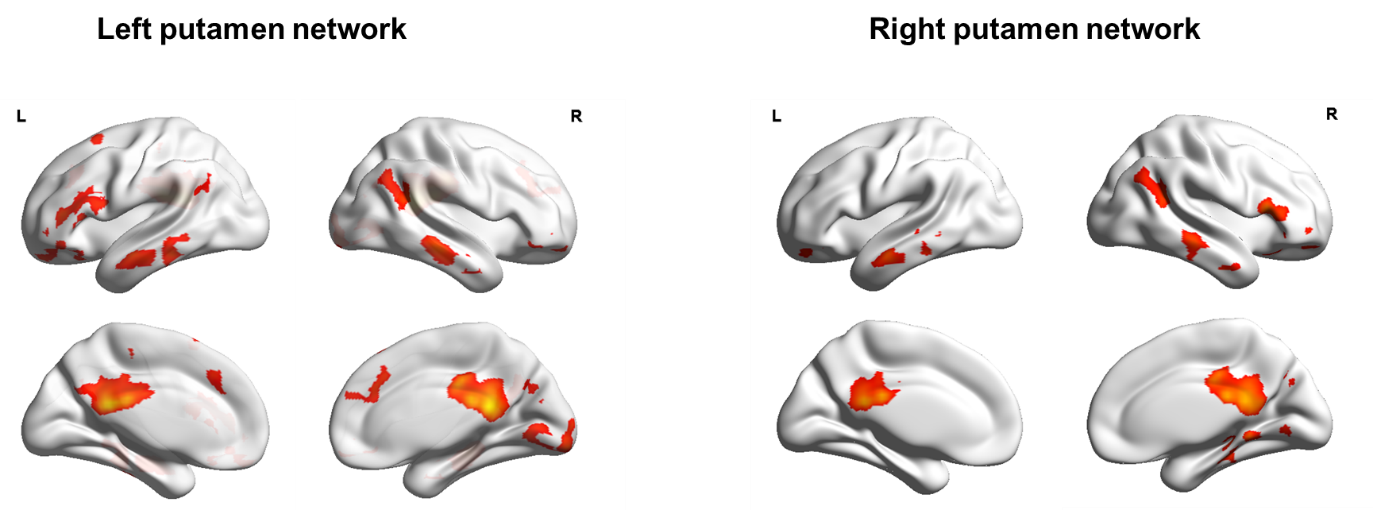

Supplement: Supplementary file 1 — Supplementary Information. [file 41598_2023_33634_MOESM1_ESM.docx]
